# Non-invasive ventilation in the treatment of early hypoxemic respiratory failure caused by COVID-19: considering nasal CPAP as the first choice

**DOI:** 10.1186/s13054-020-03054-7

**Published:** 2020-06-11

**Authors:** Lili Guan, Luqian Zhou, Jehane Michael Le Grange, Zeguang Zheng, Rongchang Chen

**Affiliations:** 1 State Key Laboratory of Respiratory Disease, National Clinical Research Center for Respiratory Disease, Guangzhou Institute of Respiratory Health, The First Affiliated Hospital of Guangzhou Medical University, Guangzhou, Guangdong China; 2 Department of Emergency Medicine, Union Hospital, Tongji Medical College, Huazhong University of Science and Technology, Wuhan, Hubei China; 3 Department of Respiratory and Critical Care Medicine, First Affiliated Hospital of Southern University of Science and Technology, Second Clinical Medical College of Jinan University, Shenzhen People’s Hospital, Shenzhen Institute of Respiratory Diseases, 1017 Dong Men Road, Shenzhen, 518020 China

High-flow nasal oxygen (HFNO) and non-invasive ventilation (NIV) have been used to manage early acute hypoxemic respiratory failure (AHRF) caused by COVID-19. As there is no evidence-based recommendation for the selection of HFNO or NIV, staff tend to base their choice on personal preference (Fig. 1).

**Fig. 1:**
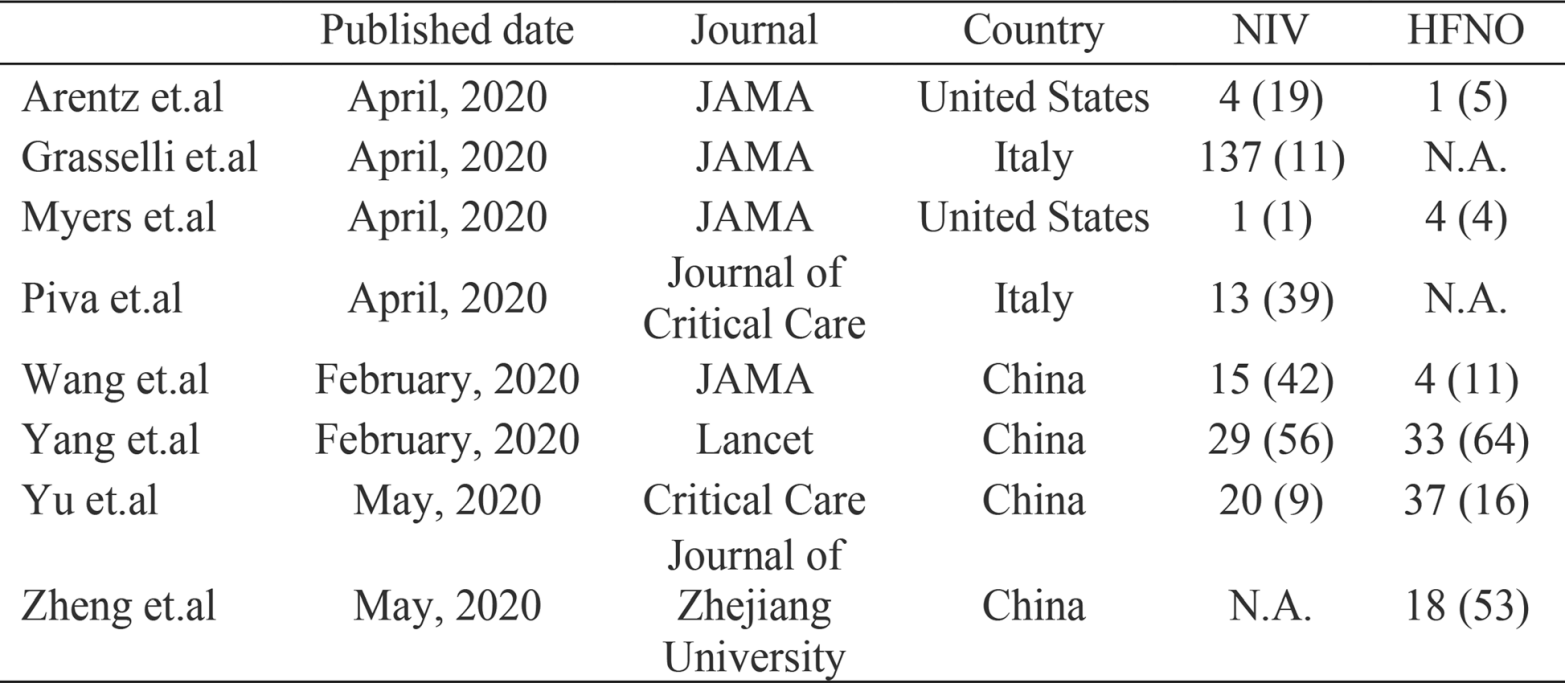
Proportion of patients with COVID-19 receiving NIV or HFNO in ICU among different studies. Data are *n* (%). COVID-19, coronavirus disease 2019; NIV, non-invasive ventilation; HFNO, high flow nasal oxygen; ICU, intensive care unit, N.A., not applicable

Frat et al. [1] showed that HFNO was associated with lower 90-day mortality in AHRF patients, which had a strong impact on clinical practice. However, there are some limitations in methodology. Firstly, NIV median daily usage was only 8 h. Furthermore, high expiratory tidal volume (9.2 ± 3.0 mL/kg) and low PEEP (5 cmH_2_O) may have negative impact on the efficacy of NIV. When considering therapeutic mechanisms, adjustable airway pressure, oxygen consumption, and patient tolerance, nasal continuous positive airway pressure (nCPAP) seems to have advantages and should be considered as the first choice.

As for therapeutic mechanism, HFNO is supposed to generate low PEEP (3 cmH_2_O on average). However, this pressure level is unstable, uncontrollable, and affected by many factors [2]. In contrast, nCPAP can provide stable and adjustable airway pressure.

When considering constant, high fraction of inspired oxygen (FiO_2_) and oxygen consumption, HFNO has the advantage of providing stable FiO_2_. However, it consumes large amounts of oxygen. When FiO_2_ is set to be 50% and flow to be 50 L/min, 18.4 L/min of 100% oxygen will be consumed. With nCPAP, a mean of 50% FiO_2_ can be achieved with 5–6 L/min of 100% oxygen delivered directly into the mask. Given current resource limitations, oxygen supply should be an important consideration as patients requiring oxygen increases dramatically.

Patient tolerance when continuously using HFNO or NIV is another consideration, as continuous positive airway pressure without interruption seems important during AHRF, especially early ARDS [3]. HFNO has particular advantage in tolerance. However, nCPAP remains well-tolerated with no patient-ventilator asynchrony.

With regard to concerns that nCPAP may increase risk of transmission, evidence remains controversial. Recent study stated that exhaled air dispersion would also increase during HNFO, theoretically making it no better than nCPAP [4]. In Guangdong, China, no healthcare workers were infected during NIV management under the Chinese guidance of personal protection [5].

In conclusion, there remains paucity evidence on how to choose between HFNO and nCPAP treating mild AHRF due to COVID-19. Theoretically, nCPAP has more advantages. Prospective randomized controlled trials are necessary to compare HFNO with nCPAP to provide more evidence on the indications for different non-invasive respiratory support and also indications for selecting between NIV and intubation.

## Data Availability

Not applicable.

## Abbreviations

HFNO: High-flow nasal oxygen
NIV: Non-invasive ventilation
AHRF: Acute hypoxemic respiratory failure
COVID-19: Coronavirus disease 2019
nCPAP: Nasal continuous positive airway pressure
FiO_2_: Fraction of inspired oxygen

## References

[1] Frat JP, Thille AW, Mercat A, Girault C, Ragot S, Perbet S, Prat G, Boulain T, Morawiec E, Cottereau A (2015). High-flow oxygen through nasal cannula in acute hypoxemic respiratory failure. N Engl J Med.

[2] Parke RL, Eccleston ML, McGuinness SP (2011). The effects of flow on airway pressure during nasal high-flow oxygen therapy. Respir Care.

[3] Ferrer M, Esquinas A, Leon M, Gonzalez G, Alarcon A, Torres A (2003). Noninvasive ventilation in severe hypoxemic respiratory failure: a randomized clinical trial. Am J Respir Crit Care Med.

[4] Leonard S, Atwood CW Jr, Walsh BK, DeBellis RJ, Dungan GC, Strasser W, Whittle JS. Preliminary findings on control of dispersion of aerosols and droplets during high-velocity nasal insufflation therapy using a simple surgical mask: implications for the high-flow nasal cannula. Chest. 2020. 10.1016/j.chest.2020.03.043. 10.1016/j.chest.2020.03.043 PMC7130245 32247712

[5] Respiratory Care Committee of Chinese Thoracic S (2020). Expert consensus on preventing nosocomial transmission during respiratory care for critically ill patients infected by 2019 novel coronavirus pneumonia. Zhonghua jie he he hu xi za zhi.

